# Genome-wide analysis of the hypoxia-related DNA methylation-driven genes in lung adenocarcinoma progression

**DOI:** 10.1042/BSR20194200

**Published:** 2020-02-20

**Authors:** Hongxia Li, Li Tong, Hong Tao, Zhe Liu

**Affiliations:** Department of Medical Oncology, Beijing Chest Hospital, Capital Medical University, Beijing Tuberculosis and Thoracic Tumor Research Institute, Beijing 101149, China

**Keywords:** DNA methylation, Hypoxia-related gene, Hypoxia, Lung adenocarcinoma

## Abstract

Lung adenocarcinoma (LUAD) is a common type of lung cancer with high incidence and poor prognosis. Hypoxia and DNA methylation play important regulatory roles in cancer progression. The purpose of the present study was to explore the relationship between hypoxia and DNA methylation, and to identify key genes for hypoxia-regulated LUAD progression. Hypoxia score (HS) was calculated using the GSVA algorithm. Gene Ontology (GO), Kyoto Encyclopedia of Genes and Genomes (KEGG) pathway enrichment and protein–protein interaction (PPI) analysis were performed using clusterProfile package, STRING database and Cytoscape software. Kaplan–Meier curves of overall survival (OS) and disease-free survival (DFS) were drawn using R software. Smoking status and cancer stages were significantly associated with LUAD hypoxia, and hypoxia is a poor prognostic factor for LUAD. Compared with HS-low group, 1803 aberrantly methylated DEGs were identified in HS-high group. KEGG analysis showed that the 1803 genes were enriched in the metabolic pathways associated with hypoxia stress, angiogenesis and cancer progression. FAM20C, MYLIP and COL7A1 were identified as the hypoxia-related key genes in LUAD progression, which were regulated by DNA methylation. Hypoxia in LUAD tumor cells led to changes in DNA methylation patterns. In-depth study of the relationship between hypoxia and DNA methylation is helpful to elucidate the mechanism of tumorigenesis, and provides new ideas for LUAD treatment.

## Introduction

Non-small cell lung cancer is a common malignant tumor worldwide, accounting for almost 85% of the total number of lung cancers [1]. Lung adenocarcinoma (LUAD) is an important pathological type of non-small cell lung cancer, and the incidence of LUAD is increasing year by year. Lack of early diagnosis system and effective treatment, tumor recurrence and chemoresistance are the main problems in the diagnosis and treatment of lung cancer [2,3]. Therefore, further exploring the biological mechanism of lung cancer progression, establishing an early detection system, identifiying key therapeutic targets and overcoming chemotherapy resistance are the keys to improving the outcome of patients with NSCLC.

Hypoxia is a characteristic microenvironment of solid tumors, and is closely related to the occurrence and development of tumors [4–6]. The hypoxic regions in human malignant parenchymal tumors have important effects on tumor biological behavior [7]. Hypoxic tumor cells promote angiogenesis by altering their metabolism to ensure tumor cell survival. In addition, hypoxia can also induce changes in the tumor microenvironment [7,8]. Hypoxia induces the expression of matrix metalloproteinases and other protease genes through hypoxia-inducible factors (HIFs), which leads to degradation of the matrix surrounding the tumor and provides a “green” channel for tumor metastasis [9]. Furthermore, hypoxia microenvironment can also affect the sensitivity of tumor cells to treatment [10]. However, the role of the hypoxia in the development of LUAD is still unclear.

In addition to genetic abnormalities, tumor cells are also regulated by epigenetic mechanisms such as local hypermethylation of CpG islands and extensive hypomethylation of the genome [11,12]. However, the mechanism that causes changes in DNA methylation patterns remains unclear. Many researches have confirmed that epigenetic changes in many genes are closely related to tumorigenesis, prognosis and drug resistance [13–15]. DNA methylation is negatively correlated with gene expression, and regulates gene function by affecting the spatial conformation of chromatin. In addition, DNA methylation can inhibit gene expression by directly inhibiting polymerases activities [16]. Hypoxia has been confirmed to be closely related to the genetic instability of tumor cells, and plays important roles in the occurrence and development of tumors [4–6]. However, the relationship between hypoxia and abnormal DNA methylation of tumor cells during the development of LUAD remains to be further explored.

## Materials and methods

### Data sets

The clinical information, gene expression and DNA methylation profiles of 533 LUAD patients in The Cancer Genome Atlas (TCGA) database (https://tcga-data.nci.nih.gov/tcga/) were downloaded. Hypoxia score (HS) was analyzed according to the hypoxia system-related metagene clusters and a gene set variation analysis (GSVA) [17,18]. One-Way ANOVA analysis was used to assess the differences of HS in different smoking states or different tumor stages.

### Identification of differentially expressed genes (DEGs) and differentially methylated genes (DMGs)

*P* < 0.05 and |log2(fold change)|>2.0 were considered as the cutoff values for DMGs and DEGs identification. The pheatmap package of R software was used to generate heat map. Distribution analysis of differentially methylated probes (DMPs) was performed according to the previous reference [19].

Hypomethylated-upregulated genes were identified by overlapping the hypomethylated genes and up-regulated genes. Hypermethylated-downregulated genes were identified by overlapping the hypermethylated genes and down-regulated genes.

### Gene Ontology (GO), Kyoto Encyclopedia of Genes and Genomes (KEGG), protein–protein interaction (PPI) network and motif enrichment analysis

GO and KEGG analysis of the aberrantly methylated DEGs were carried out using clusterProfile package, with *P* < 0.05 as the screening standard. For PPI network analysis, the aberrantly methylated DEGs were analyzed by STRING database (version 11.0) (https://string-db.org/), and then the results were screened by Molecular Complex Detection (MCODE) in Cytoscape software (http://www.cytoscape.org) with default parameter. The 2 -kb upstream region of FAM20C, MYLIP and COL7A1 promoters was further analyzed with Transcription factor Affinity Prediction (TRAP) Web Tools to identify enriched motifs [20].

### Survival analysis

To analyze the effect of hypoxia on the prognosis, data in TCGA database were divided into HS-high group and HS-low group according to the hypoxia score, and Kaplan–Meier curves of overall survival (OS) and disease-free survival (DFS) were drawn using R software. In order to analyze the effects of genes expression on the patient’s OS, Kaplan–Meier analysis was performed based on the data in TCGA database. Genes with significant differences of OS between the high-expression group and low-expression group were screened, and then the screened genes were verified using the data downloaded from Kaplan Meier-plotter database (http://www.kmplot.com). Similarly, Kaplan–Meier curves were plotted based on the data of DNA methylation probes in TCGA database to analyze the effects of DNA methylation status on the prognosis. *P* < 0.05 was accepted as significant difference.

### Cell culture and cell treatment

Human lung adenocarcinoma cell line, A549, was purchased from ATCC and cultured in DMEM medium (Gibco, Carlsbad, CA, U.S.A.) with 10% FBS (Gibco) at 37°C with 5% CO_2_. For hypoxia treatment, cells were cultured in tri-gas incubator (Thermo, MA, U.S.A.) consisting of 2% O_2_, 5% CO_2_ and 93% N_2_ for 24 h. Then, the cells were treated with the DNA methylation inhibitor 5-Aza-2′-deoxycytidine (Aza) (10 μM, Sigma, U.S.A.).

### Quantitative real-time PCR (qRT-PCR)

The total RNA of cells were extracted using TRIzol reagent (Invitrogen). PrimeScript RT reagent kit (Takara, Japan) and SYBR Premix Ex Taq II (Takara) were applied for reverse transcription and qRT-PCR, respectively. GAPDH was selected as internal reference gene. The relative expression levels of mRNA were calculated using 2−ΔΔ*C*t method. Primers used in the present study were provided in Table 1.

**Table 1:** Primers used for qRT-PCR analysis

Genes	Primer sequences (5*′*-3*′*)
FAM20C	F: 5*′*- GCCGTGGACTCCTATCCCA-3*′*
	R: 5*′*- GCCCGTAATTCTGGAAGGTCA-3*′*
MYLIP	F: 5*′*- GCAGGCGACTGGGAATCATAG -3*′*
	R: 5*′*- CGGTTTCTCAGGTTTAGCCAT-3*′*
COL7A1	F: 5*′*- TTACGCCGCTGACATTGTGTT-3*′*
	R: 5*′*- ACCAGCCCTTCGAGAAAGC-3*′*
GAPDH	F: 5*′*- TGTGGGCATCAATGGATTTGG-3*′*
	R: 5*′*- ACACCATGTATTCCGGGTCAAT-3*′*

## Results

### Relationship between hypoxia and smoking, cancer stage or prognosis

**Figure 1:**
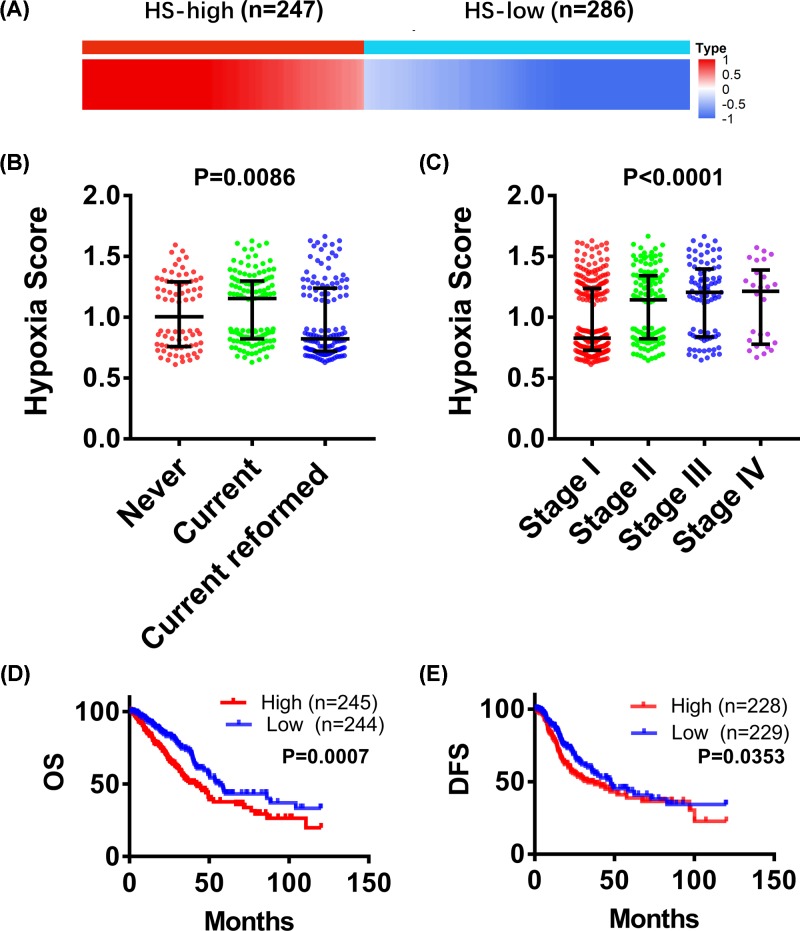
Relationship between hypoxia and smoking, cancer stage and prognosis (**A**) 533 LUAD patients were divided into two groups: hypoxia score (HS)-high group (*n* = 247) and HS-low group (*n* = 286), according to their HS values. One-way ANOVA analysis was performed to evaluate the differences of HS in different smoking states or different tumor stages. (**B**) HS of LUAD patients who reformed smoking, no-smoking and smoking. (**C**) HS of different cancer stages. (**D**) Analysis of overall survival (OS) based on HS. HS-high group, *n* = 245; HS-low group, *n* = 244. (**E**) Analysis of disease-free survival (DFS) based on HS. HS-high group, *n* = 228; HS-low group, *n* = 229.

A total of 533 patients with LUAD were divided into HS-high group (*n* = 247) and HS-low group (*n* = 286) according to their HS values. As shown in Figure 1B, the HS values of LUAD patients who reformed smoking (average HS = 0.8), never-smoking (average HS = 1.0) and smoking (average HS = 1.16) increased significantly in turn. In addition, the average HS increased with the increase of cancer stages (*P* < 0.0001) (Figure 1C). Then, the effects of hypoxia on prognosis were analyzed. Kaplan–Meier survival curves suggested that both OS and DFS of HS-high group were notably lower than those of HS-low group (Figure 1C,D). Taken together, these results revealed that smoking status and cancer stages were significantly associated with LUAD hypoxia, and patients with a higher degree of hypoxia had a poorer outcome.

### Aberrantly methylated DEGs in HS-high group compared with HS-low group

**Figure 2:**
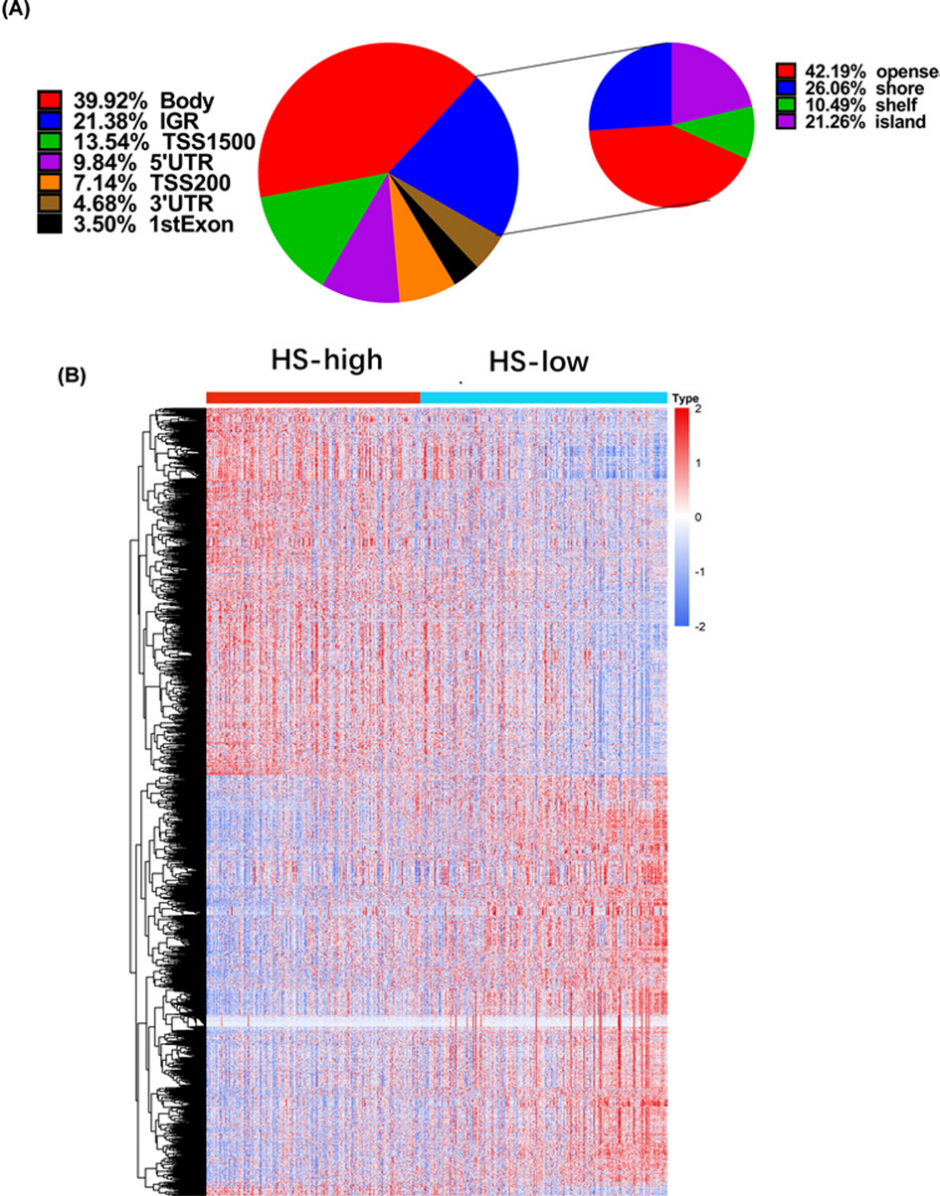
Analysis of DMGs and DEGs in HS-high group compared with HS-low group (**A**) Distribution of the DMPs with different genomic feature. (**B**) Heat map of DEGs between HS-high group and HS-low group. *P* < 0.05 and |log2 (fold change) |>2.0 were selected as the cutoff values.

To explore the effect of hypoxia degree on DNA methylation status of LUAD cells, a total of 12176 DMGs were identified according to *P* < 0.05 and |log2(fold change) |>2.0. Subsequently, the DMPs were classified according to the genomic feature (Figure 2A). The DMPs mainly located in gene body (39.92%) and noncoding intergenic region (IGR, 21.38%) (Figure 2A). Remarkably, the distribution frequency of DMPs in open sea was the highest in IGR (Figure 2A). Five thousand and sixty five DEGs were identified, including 2413 up-regulated genes and 2652 down-regulated genes (Figure 2B).

**Figure 3:**
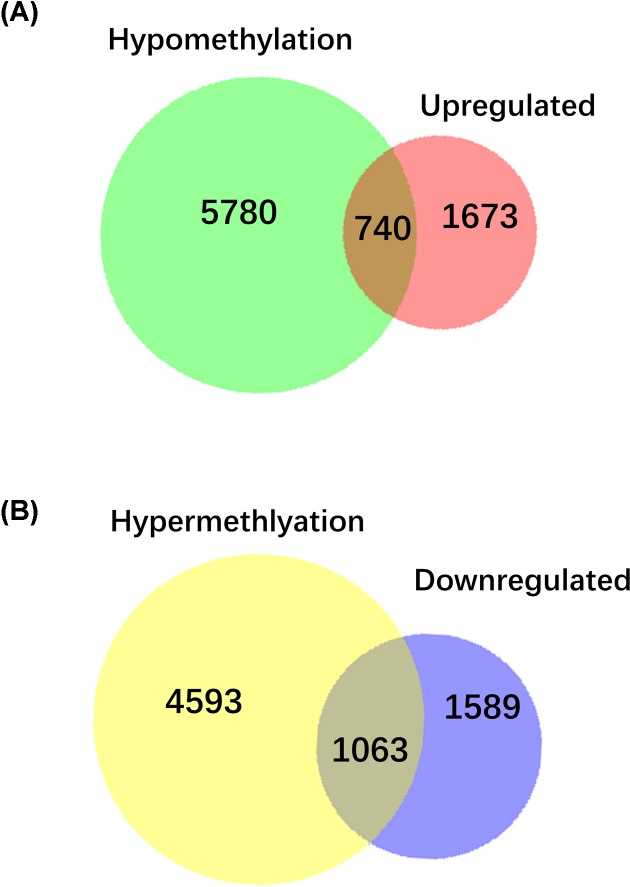
Overlapping relationship between DEGs and DMGs (**A**) Venn diagram of hypomethylation and up-regulated genes. (**B**) Venn diagram of hypermethylation and down-regulated genes.

Venn diagram showed that there were 740 hypomethylated-upregulated genes were overlapped between hypomethylated DMGs (*n* = 6520) and up-regulated DEGs (*n* = 2413) (Figure 3A). A total of 1063 hypermethylated-downregulated genes were overlapped between hypermethylated DMGs (*n* = 5656) and down-regulated DEGs (*n* = 2652) (Figure 3B). Collectively, a total of 1803 aberrantly methylated DEGs (including 740 hypomethylated-upregulated genes and 1063 hypermethylated-downregulated genes) were identified in HS-high group compared with HS-low group.

### GO term and KEGG pathway analysis

**Figure 4:**
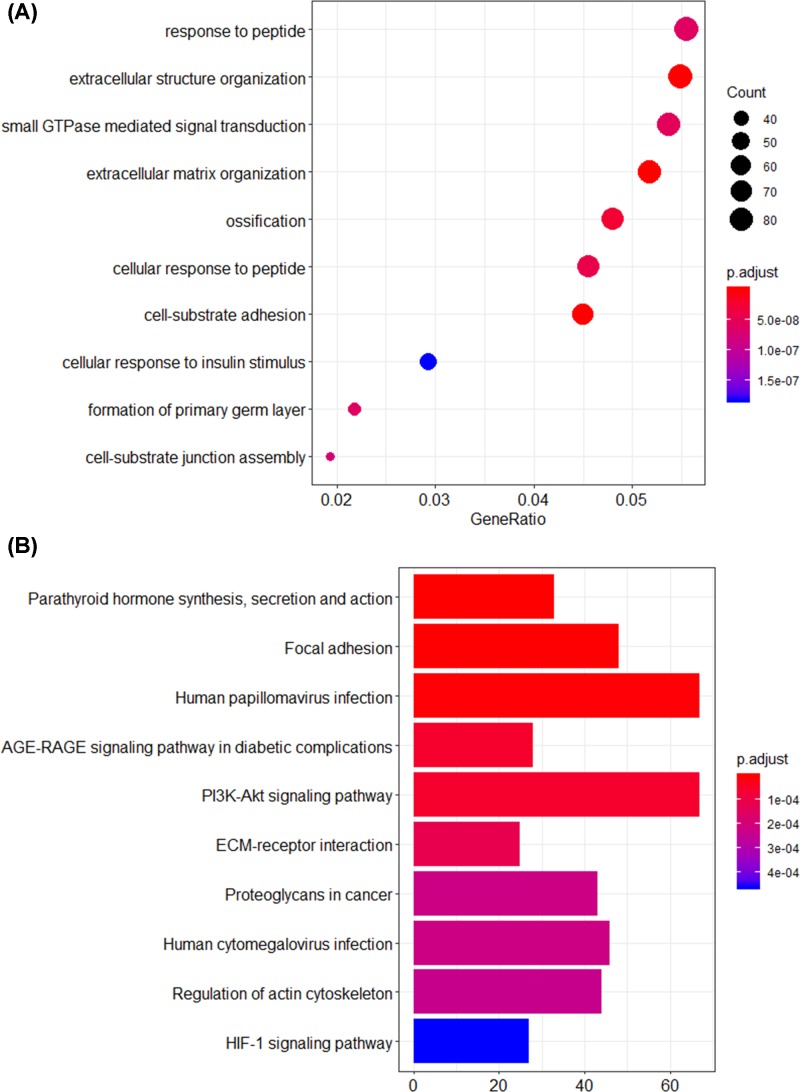
GO term and KEGG pathway analysis for the aberrantly methylated DEGs (**A**) The top 10 significant GO terms. (**B**) The top 10 significant KEGG pathways.

To further investigate the effects of DNA methylation status on the above overlapped genes, all of the 1803 aberrantly methylated DEGs were used for GO and KEGG analysis. The top 10 significant GO terms were presented in Figure 4A. GO analysis showed that the aberrantly methylated DEGs were significantly enriched in response to peptide, extracellular structure organization and small GTPase mediated signal transduction. Furthermore, KEGG analysis suggested that the metabolic pathways associated with hypoxia stress, angiogenesis and cancer progression were enriched such as PI3K-Akt signaling pathway and HIF-1 signaling pathway (Figure 4B).

### PPI analysis

**Figure 5:**
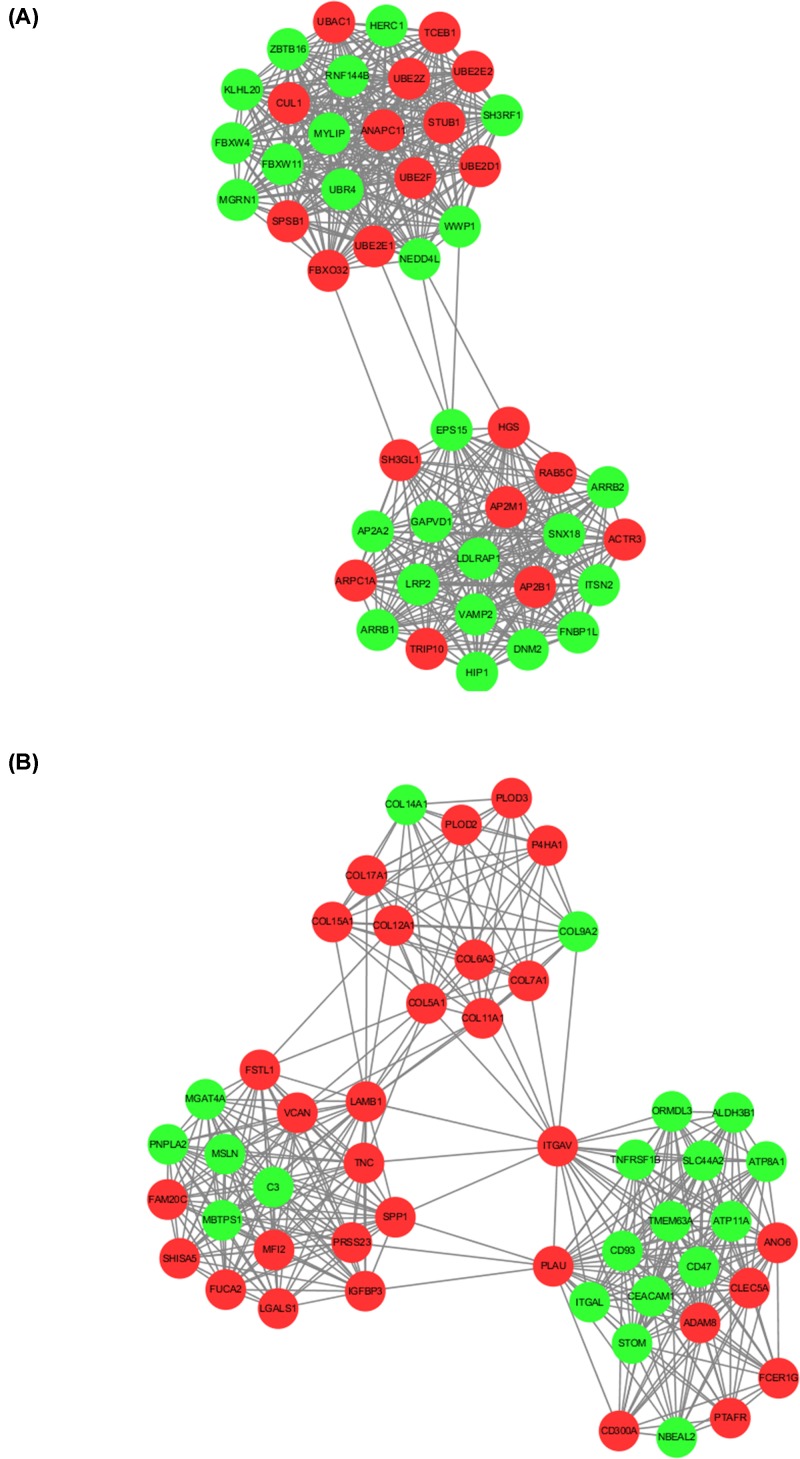
The top 2 significant modules in PPI network Red nodes: up-regulated gene in HS-high group versus HS-low group; Green nodes: down-regulated gene in HS-high group versus HS-low group.

All of the 1803 aberrantly methylated DEGs were analyzed by STRING database and screened by Cytoscape software. The PPI network consisted of 32 modules, containing a total of 1692 nodes and 14,422 edges. The top 2 significant modules were shown in Figure 5. There were 45 nodes and 491 edges in the module 1 (Figure 5A). A total of 407 edges and 50 nodes formed the module 2 (Figure 5B).

### Survival analysis of the enriched genes in modules 1 and 2 of PPI network

**Figure 6:**
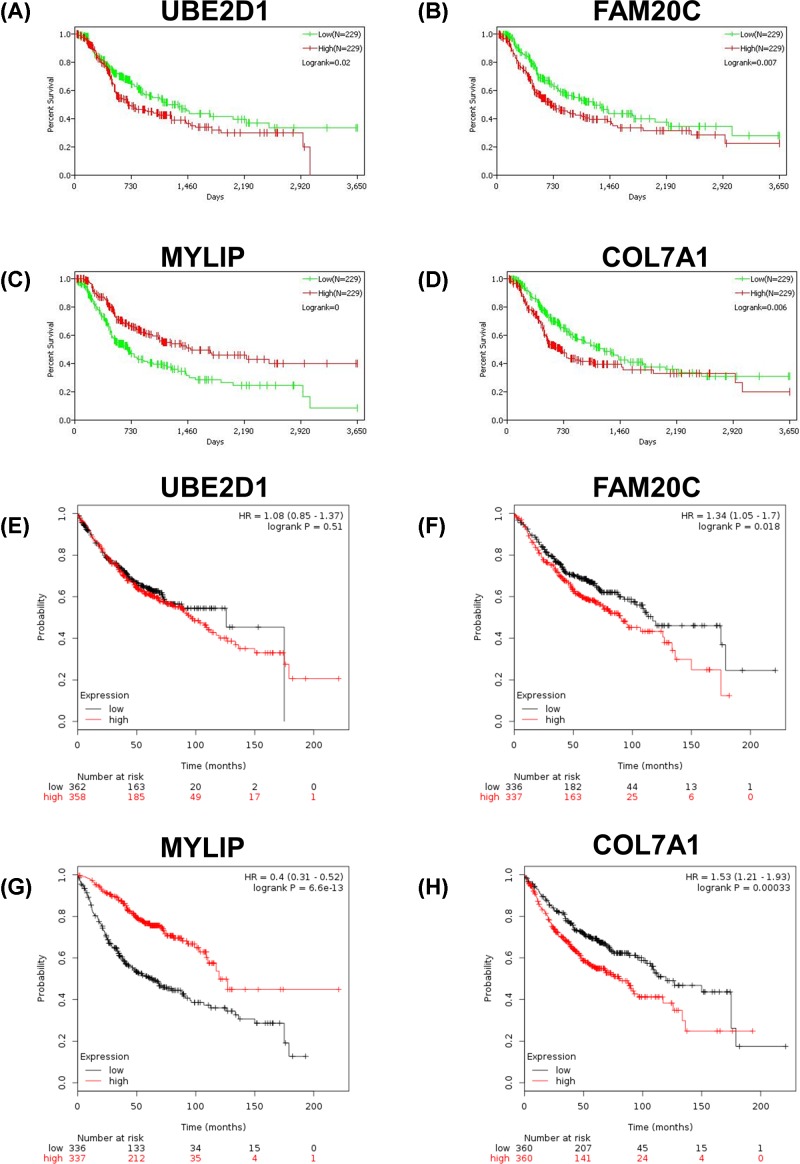
Survival analysis of UBE2D1, FAM20C, MYLIP and COL7A1 expression (**A–D**) UBE2D1 (A), FAM20C (B), MYLIP (C) and COL7A1 (D) in TCGA database. (**E–H**) UBE2D1 (E), FAM20C (F), MYLIP (G) and COL7A1 (H) in Kaplan Meier-plotter database.

**Figure 7:**
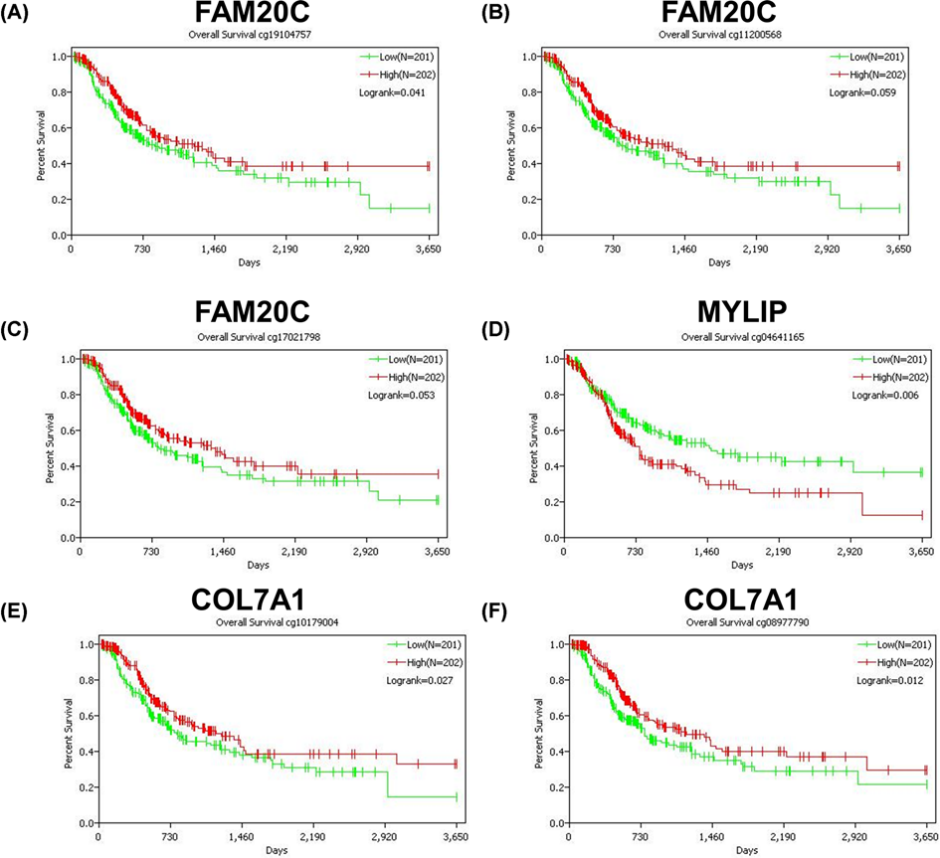
DNA methylation status of FAM20C, MYLIP and COL7A1 associated with overall survival (**A–C**) Methylation probes of FAM20C. (**D**) Methylation probe of MYLIP. (**E** and **F**) Methylation probes of COL7A1.

**Figure 8:**
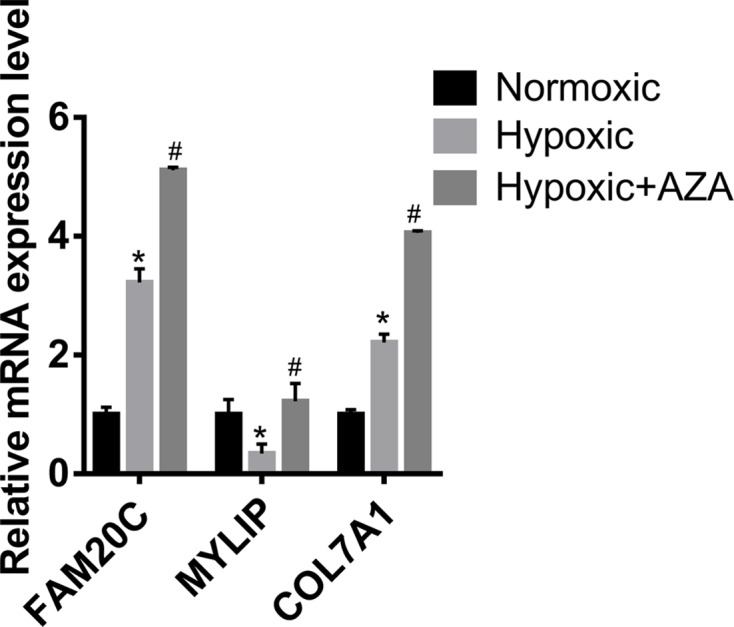
Relative expression levels of FAM20C, MYLIP and COL7A1 in normoxic, hypoxic and hypoxic+Aza groups *P < 0.05, hypoxic group versus normoxic group. #*P* < 0.05, hypoxic+Aza group versus hypoxic group.

To further identify the key genes related to hypoxia in LUAD, survival analysis of the 95 enriched genes (including 45 genes in module 1 and 50 genes in module 2) in TCGA database was performed. The data suggested that the expression levels of UBE2D1, FAM20C, MYLIP and COL7A1 were significantly associated with the prognosis of LUAD (Figure 6A–D). Subsequently, the survival analysis of UBE2D1, FAM20C, MYLIP and COL7A1 were validated in the Kaplan Meier-plotter database. High expression levels of FAM20C and COL7A1 were significantly associated with poor prognosis (Figure 6F,H), while patients with high expression level of MYLIP had a better prognosis (Figure 6G). The above results were consistent with the results of TCGA database. However, the survival analysis of data in Kaplan Meier-plotter database showed that UBE2D1 expression had no significant effect on the prognosis of LUAD (Figure 6E). Finally, data of methylation probes of FAM20C, MYLIP and COL7A1 in TCGA database were downloaded. Hypomethylation of FAM20C (Figure 7A–C) and COL7A1 (Figure 7E and F) was associated with poor outcome, while the hypomethylation of MYLIP (Figure 7D) was associated with better survival. To verify FAM20C, MYLIP and COL7A1 were the hypoxia-related DNA methylation-driven genes, A549 cells were divided into normoxic, hypoxic and hypoxic+Aza groups. As shown in Figure 8, hypoxia treatment significantly increased FAM20C and COL7A1 expression, and DNA methylation inhibitor Aza treatment futher promoted the expression of FAM20C and COL7A1. MYLIP was down-regulated in hypoxic group compared with normoxic group, while Aza treatment reversed this effect (Figure 8). Taken together, FAM20C, MYLIP and COL7A1 were identified as the key potential genes affecting hypoxia-associated LUAD progression.

### FAM20C, MYLIP and COL7A1 potentially targeted by NF-κB

**Figure 9:**
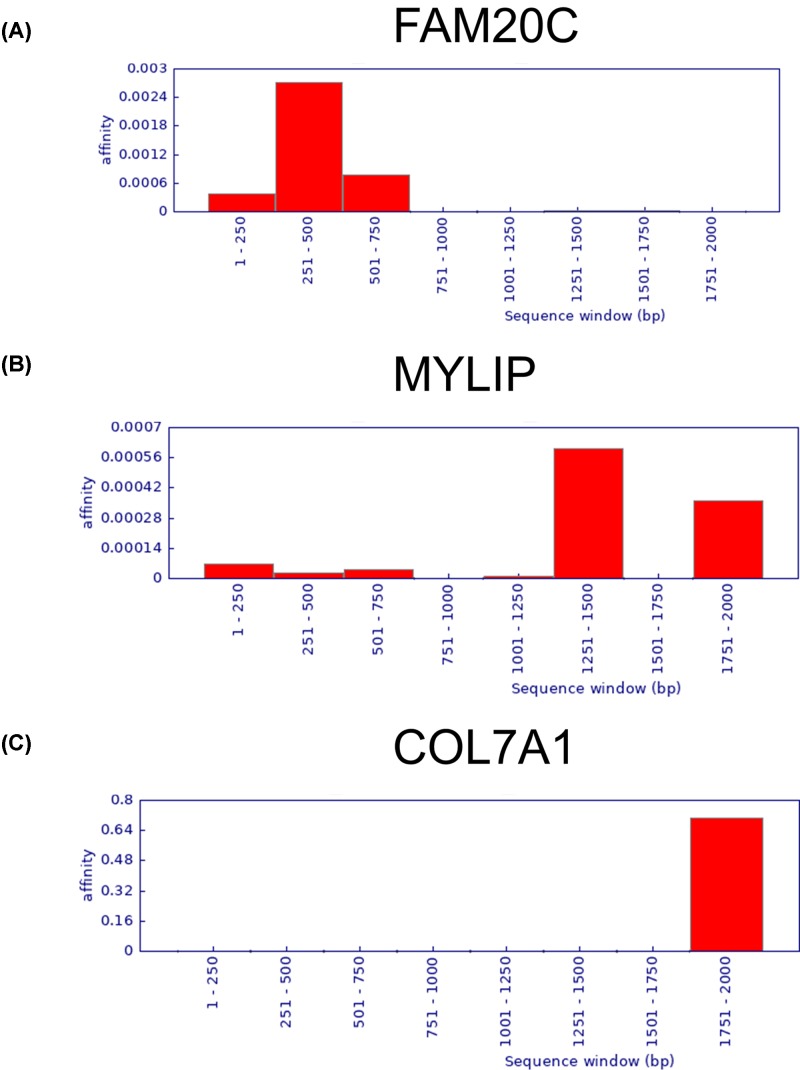
FAM20C (**A**), MYLIP (**B**) and COL7A1 (**C**) potentially targeted by NF-κB

As transcription factors hypoxia-inducible factor-1α (HIF-1α) and nuclear factor-κB (NF-κB) play a key role in the regulation of hypoxia and tumorigenesis, we analyzed the 2 -kb upstream region of FAM20C, MYLIP and COL7A1 promoters for the HIF-1α and NF-kB-binding sequence. Indeed, FAM20C, MYLIP and COL7A1 are potentially regulated by NF-kB. However, we did not find a binding site for HIF-1α (Figure 9). These results suggest that NF-kB may directly transactivates the expression of FAM20C, MYLIP and COL7A1 during hypoxia-associated LUAD progression.

## Discussion

The hypoxic microenvironment is an important feature of solid tumors [4–6]. In the present study, we demonstrated a positive correlation between hypoxia degree and tumor stages in LUAD, and patients with high degree of hypoxia had poor prognosis. Under hypoxic conditions, tumor cells secrete a variety of angiogenic factors to promote the abnormal angiogenesis. In addition, hypoxia improves the invasion and metastasis ability of tumor cells, increases the malignancy of tumor, and further results in insensitivity of tumor cells to chemotherapy drugs or radiation therapy [7,8]. Thus, the hypoxic microenvironment is an important factor in cancer poor prognosis.

Tumor development is often accompanied by DNA methylation imbalance. The inactivation of tumor suppressor genes caused by abnormal hypermethylation is particularly important [21]. Studies have shown that hypoxia is one of the important driving forces for DNA methylation imbalance in tumor cells, and interacts with epigenetic regulation mechanisms to promote tumor development [22,23]. In the present study, a total of 12176 DMGs were identified by comparing DNA methylation status in HS-high and HS-low groups. The results reminded that hypoxia can cause extensive methylation imbalance in tumor cells. This phenomenon may be related to the down-regulation of DNA methyltransferases, and the regulation of histone modification and DNA methylation patterns [24,25].

The epigenetic regulation can further promote the hypoxic adaptive response, help tumor cells acquire more malignant phenotypes, and escape the toxic effects of radiotherapy and chemotherapy [26,27]. The prsent study explored the key genes involved in the development of hypoxia-associated LUAD, and their interactions with other factors. We identified 1803 hypoxia-related aberrantly methylated DEGs, and then performed annotation and functional analysis of these genes. We found that hypoxia-related aberrantly methylated DEGs were significantly involved in hypoxia stress, angiogenesis and cancer progression related biological processes. The skeletal system is one of the most common sites of lung cancer metastasis [28,29]. Ossification is associated with LUAD bone metastasis, and the hypoxic microenvironment plays important roles in this process [30,31]. HIFs is an important class of oxygen-dependent transcriptional activators. The expression of HIFs can promote the adaptation of tumor cells to hypoxia microenvironment [9]. Moreover, HIFs can further regulate a variety of genes related to invasion and metastasis of tumor cells [32,33]. The high expression of HIFs can be considered as a high risk factor of metastasis. Other biological pathways enriched in the present study, such as proteoglycans in cancer, PI3K-Akt signaling pathway, extracellular structure organization and small GTPase mediated signal transduction, were also closely related to tumor progression and hypoxic microenvironment formation. These results indicated that hypoxia can cause imbalance of DNA methylation in tumor cells, and further affect tumor progression and metastasis.

To further screen the key genes in the hypoxia-related LUAD progression, we constructed a PPI network and performed prognostic analysis. FAM20C, MYLIP and COL7A1 were identified as the key potential genes affecting hypoxia-related LUAD progression. FAM20C is an intracellular casein kinase that phosphorylates hundreds of secreted proteins to regulate a variety of physiological and pathological processes, including skeletal development and blood phosphorus metabolism. Studies have shown that FAM20C regulates the redox homeostasis of the endoplasmic reticulum by phosphorylating the endoplasmic reticulum thiol oxidase Ero1a during hypoxic stress [34]. However, the relationship between FAM20C and LUAD has not been reported. In the present study, we found that hypoxia in LUAD cells inhibited DNA methylation of FAM20C gene, promoted FAM20C gene expression, and further led to deterioration of LUAD. The expression levels and methylation status of FAM20C can be used as markers for judging the prognosis of LUAD. Myosin-regulated light chain interacting protein (MYLIP), also known as ubiquitin–protein ligase, regulates the post-transcriptional diversity of low-density lipoprotein (LDL) receptors. Studies have confirmed that MYLIP inhibits the metastasis and progression of breast cancer [35]. Our study indicated that MYLIP was a tumor suppressor during the progression of LUAD, and hypoxia promoted DNA methylation of MYLIP. COL7A1 encoding type VII collagen. Collagen changes in the tumor microenvironment are mainly manifested in nascent collagen, density, direction, length, cross-linking and so on. These changes affect tumor cell metabolism, macromolecular transport, gene expression, angiogenesis, and also affect tumor invasion and metastasis by regulating epithelial–mesenchymal transition, immunity and stromal cells [36]. Our study confirmed that COL7A1 was an oncogene of LUAD and was associated with poor prognosis of LUAD. Hypoxia led to hypomethylation of COL7A1.

Hypoxic microenvironment will change the metabolism of tumor cells, induce adaptive changes in cell metabolism, and regulate complex cellular signaling pathways such as HIF-1α and NF-κB [37]. Among them, NF-κB participates in inflammatory response and regulates cell proliferation and survival [38–40]. At the same time, NF-κB can also regulate the expression of key mediators of endothelial cell survival and angiogenesis such as AKT and VEGF [41,42]. Our study showed that NF-kB may directly transactivates the expression of FAM20C, MYLIP and COL7A1 during hypoxia-associated LUAD progression.

In conclusion, we performed a genome-wide comparative analysis on the hypoxia-related DNA methylation profiles of LUAD based on TCGA database. The results reminded that hypoxia in LUAD tumor cells led to changes in DNA methylation patterns. FAM20C, MYLIP and COL7A1 were identified as the hypoxia-related key genes in LUAD progression, which were regulated by DNA methylation. In-depth study of the relationship between hypoxia and DNA methylation is helpful to clarify the mechanism of tumorigenesis, and provides new ideas for LUAD treatment.

## Data Availability

All relevant data are contained within the paper. Additional information can be obtained by contacting Dr Zhe Liu (zheliu_lza@163.com).

## Abbreviations

DEG: differentially expressed gene
DFS: disease-free survival
DMG: differentially methylated gene
DMP: ifferentially methylated probe
GO: Gene Ontology
HIF: hypoxia-inducible factor
HS: hypoxia score
KEGG: Kyoto Encyclopedia of Genes and Genomes
LUAD: lung adenocarcinoma
OS: overall survival
PPI: protein–protein interaction
